# Supplementary material for: Epidemiology and control of maedi-visna virus: Curing the flock
Source: PLoS One. 2020 Sep 10;15(9):e0238781. doi: 10.1371/journal.pone.0238781 (PMC7482963; doi:10.1371/journal.pone.0238781)
Supplement: S3 File — Derivation of the mathematical model, selection of prior distributions, derivation of R0 and prevalence doubling time and assessment of Markov chains of the optimal model. (PDF) [file pone.0238781.s003.pdf]

Supplementary Material: Epidemiology and control of maedi-visna virus: curing the flock

Andrew W. Illius, Karianne Lievaart-Peterson, Tom N. McNeilly and Nicholas J. Savill

1 Description of the full mathematical model

1.1 Overview of the model

Our aim is to derive equations for the probability density functions (PDFs) of seroconversion times of randomly selected susceptible ewes introduced into the flock at given times. We then use these PDFs to derive the likelihood of the observed serostatuses of experimental ewes given particular model parameter values.

The time a susceptible ewe is infected T, its seroconversion period X, and the time it seroconverts $S = T + X$, are all unknown and are therefore treated as random variables with associated probability distributions.

Let $\beta(t)$ be the per-capita transmission rate at time t after the start of the experiment (March 25th 1980), let $I(t)$ be the expected number of infectious ewes at time t and let $\lambda(t)$ be the force of infection at time t. Under the assumption that ewes mix freely $\lambda(t) = \beta(t)I(t)$. For now we assume $I(t)$ is known; its derivation is given in Section 1.3.

Consider a susceptible ewe introduced into the flock at time t_0. The rate of change of its probability of being uninfected at time t, $\Pr(T > t)$, is (Cox and Miller, 1965)

$$\frac{d}{dt}\Pr(T > t) = -\lambda(t)\Pr(T > t) \tag{1}$$

The solution of this differential equation is

$$\Pr(T > t) = \exp\left(-\int_{t_0}^{t} \lambda(\xi)\, d\xi\right) \tag{2}$$

where ξ is a dummy integration variable. The cumulative distribution function (CDF) of the susceptible ewe's infection time, T, is $F_T(t; t_0) = \Pr(T \leq t)$. By definition, $\Pr(T \leq t) = 1 - \Pr(T > t)$, therefore, the CDF of T is

$$F_T(t; t_0) = 1 - \Pr(T > t) \tag{3}$$

Let $f_T(t; t_0)$ be the probability density function of the ewe's infection time. It is given by the derivative of its cumulative distribution function,

$$f_T(t; t_0) = \frac{d}{dt}F_T(t; t_0) \tag{4}$$

Substituting in Equation 3 gives

$$= -\frac{d}{dt}\Pr(T > t) \tag{5}$$

Substituting in Equation 1 gives

$$= \lambda(t)\Pr(T > t) \tag{6}$$

And substituting in Equation 2 results in

$$= \lambda(t)\exp\left(-\int_{t_0}^{t} \lambda(\xi)\, d\xi\right) \tag{7}$$

We assume a Gamma distributed seroconversion period. However, we also assume that the seroconversion rate in winter 1982, when ewes were in poor condition, may be different than at all other times when ewes were healthy. The CDF of a Gamma distributed random variable X is $F_X(x) = \Pr(X \le x) = \frac{\gamma(a,bx)}{\Gamma(a)}$ where $\gamma(a, bx) = \int_0^{bx} \xi^{a-1}e^{-\xi}\, d\xi$ is the lower incomplete gamma function, $\Gamma(a)$ is the gamma function, a is the shape of the Gamma distribution and b is the rate. To model a seroconversion rate, $b(t)$, that varies in time t through the experiment, we replace the term bx in the CDF with the function

$$Z(x;t) = \int_{t}^{t+x} b(\zeta)\, d\zeta \tag{8}$$

This is the cumulative seroconversion rate between times t and $t + x$. When $b(t) = b$ is constant we recover $Z(x;t) = bx$ which is independent of t as expected. Thus

$$F_X(x;t) = \frac{\gamma(a, Z(x;t))}{\Gamma(a)} \tag{9}$$

is the CDF of the seroconversion period, X, of a ewe infected at time t. With a time varying seroconversion rate seroconversion period is not Gamma distributed. However, except in winter 1982, seroconversion rate is constant and so seroconversion period will be Gamma distributed. The PDF of X is given by

$$f_X(x;t) = \frac{d}{dx}F_X(x;t) \tag{10}$$

Substituting in Equation 9 gives

$$= \frac{1}{\Gamma(a)}\frac{d}{dx}\gamma(a, Z(x;t)) \tag{11}$$

Applying the chain rule gives

$$= \frac{1}{\Gamma(a)}Z(x;t)^{a-1}e^{-Z(x;t)}\frac{d}{dx}Z(x;t) \tag{12}$$

Substituting in Equation 8 gives

$$= \frac{1}{\Gamma(a)}Z(x;t)^{a-1}e^{-Z(x;t)}\frac{d}{dx}\int_{t}^{t+x} b(\zeta)\, d\zeta \tag{13}$$

from which we obtain the final form of the PDF

$$= \frac{1}{\Gamma(a)}Z(x;t)^{a-1}e^{-Z(x;t)}b(t+x) \tag{14}$$

Let $f_S(s;t_0)$ be the distribution of seroconversion time S, which is the sum of infection time and seroconversion period, i.e., $S = T + X$. This distribution is, therefore, the convolution of the distribution of infection time T, and the distribution of seroconversion period X

$$f_S(s;t_0) = \int_{t_0}^{s} f_T(t;t_0) f_X(s-t;t)\,dt \tag{15}$$

If a ewe is known to have seroconverted sometime between times s_- and s_+, the probability of it doing so is the integral of its distribution of seroconversion time between these two times, i.e.,

$$p = \Pr(s_- < S \leq s_+) = \int_{s_-}^{s_+} f_S(s;t_0)\,ds \tag{16}$$

If a susceptible ewe never seroconverted between introduction into the flock and its last serological test at time t_r, its probability of doing so is

$$q = \Pr(S > t_r) = 1 - \Pr(t_0 < S \leq t_r) = 1 - \int_{t_0}^{t_r} f_S(s;t_0)\,ds \tag{17}$$

It now remains to solve the above integrals and to specify the number of infectious ewes $I(t)$.

1.2 Discretising time

It is highly desirable that the above integrals have analytical solutions to allow rapid (order of hours) sampling of the posterior distribution. However, given the time-varying transmission rate $\beta(t)$, and the time-varying seroconversion rate $b(t)$, the above integrals are not analytically solvable. In order to obtain analytical solutions we can discretise time into 1 month intervals (for Houwers) or 1 week intervals (for Peterson) which roughly match the sampling intervals. (To not interrupt the flow of our argument we only refer to monthly intervals, but the same arguments hold for weekly intervals.) This means we can treat the transmission and seroconversion rates as piecewise-constant such that in month i, transmission rate β_i, and seroconversion rate b_i, are constant. We also assume that the number of infectious ewes I_i, in month i is constant. This implies that the force of infection $\lambda_i = \beta_i I_i$, in month i is also constant.

We first discretise the distribution of infection time $f_T(t;m_0)$. The probability of a susceptible ewe being uninfected at time t (Eqt. 2) can be partitioned into monthly intervals. We set the units of time to be months with 1 month equalling $^{365}/_{12} = 30.4$ days. Let m_0 be the month a susceptible ewe was introduced into the flock and let $u_n(t;m_0)$ be the probability of it being uninfected at time t in month n (i.e., $t \in [n, n+1)$). This probability is given by

$$u_n(t;m_0) = \exp\left(-\int_{m_0}^{t} \lambda(\xi)\,d\xi\right) \tag{18}$$

$$= \exp\left(-\sum_{i=m_0}^{n-1} \lambda_i\right) e^{-\lambda_n[t-n]} \tag{19}$$

Define $u_{m_0,n} = \exp\left(-\sum_{i=m_0}^{n-1} \lambda_i\right)$, which is the probability of a ewe being uninfected before month n, and substituting into Eqt. 19 we obtain

$$u_n(t;m_0) = u_{m_0,n} e^{-\lambda_n[t-n]} \tag{20}$$

Let $f_{T,n}(t;m_0)$ be the piecewise distribution of infection time T, in month n. This is obtained by substituting Eqt. 20 into Eqt. 7:

$$f_{T,n}(t;m_0) = \lambda_n u_n(t;m_0) \tag{21}$$

$$= \lambda_n u_{m_0,n} e^{-\lambda_n[t-n]} \tag{22}$$

Second, we discretise the distribution of seroconversion period $f_X(x;t)$. Let $Z_{n,m}(x;t)$ be the cumulative seroconversion rate between times t and $t+x$ such that t is in month n (i.e., $t \in [n, n+1)$) and $t+x$ is in month m (i.e, $t+x \in [m, m+1),\ n \leq m$). It is given by

$$Z_{n,m}(x;t) = \int_t^{t+x} b(\xi)\, d\xi \tag{23}$$

$$= b_n[n+1-t] + \sum_{i=n+1}^{m-1} b_i + b_m[t+x-m] \tag{24}$$

which, with some rearranging, is

$$Z_{n,m}(x;t) = \sum_{i=n}^{m-1} b_i + b_n[n-t] + b_m[t+x-m] \tag{25}$$

Let $f_{X,n,m}(x;t)$ be the piecewise distribution of seroconversion period X, in month m given infection at time t in month n. This is obtained by substituting Eqt. 25 into Eqt. 14:

$$f_{X,n,m}(x;t) = \frac{b_m}{\Gamma(a)} Z_{n,m}(x;t)^{a-1} e^{-Z_{n,m}(x;t)} \tag{26}$$

Finally we discretise the the distribution of seroconversion time S. Let $f_{S,m}(s;m_0)$ be the piecewise distribution of seroconversion time S in month m ($s \in [m, m+1)$). This is given by Eqt. 15

$$f_{S,m}(s;m_0) = \int_{m_0}^{s} f_T(t;m_0) f_X(s-t;t)\, dt \tag{27}$$

partitioning the integral into 1 month intervals we obtain

$$\begin{aligned} f_{S,m}(s;m_0) = &\sum_{n=m_0}^{m-1} \int_n^{n+1} f_{T,n}(t;m_0) f_{X,n,m}(s-t;t)\, dt \\ &+ \int_m^s f_{T,m}(t;m_0) f_{X,m,m}(s-t;t)\, dt \end{aligned} \tag{28}$$

For convenience we define

$$z_{n,m}(t,s) = Z_{n,m}(s-t;t) \tag{29}$$

substituting in Eqt. 25 and defining $B_{n,m} = \sum_{i=n}^{m-1} b_i$ gives

$$z_{n,m}(t,s) = B_{n,m} + b_n[n-t] + b_m[s-m] \tag{30}$$

Substituting Eqts. 22 and 26 into Eqt. 28 and moving constants outside the integrals we obtain

$$\begin{aligned} f_{S,m}(s;m_0) = &\sum_{n=m_0}^{m-1} \frac{b_m \lambda_n u_{m_0,n}}{\Gamma(a)} \int_n^{n+1} e^{-\lambda_n[t-n]} z_{n,m}(t,s)^{a-1} e^{-z_{n,m}(t,s)}\, dt \\ &+ \frac{b_m \lambda_m u_{m_0,m}}{\Gamma(a)} \int_m^s e^{-\lambda_m[t-m]} z_{m,m}(t,s)^{a-1} e^{-z_{m,m}(t,s)}\, dt \end{aligned} \tag{31}$$

We define the indefinite integral

$$v_{n,m}(t,s) = \frac{b_m \lambda_n}{\Gamma(a)} \int e^{-\lambda_n[t-n]} z_{n,m}(t,s)^{a-1} e^{-z_{n,m}(t,s)}\, dt \tag{32}$$

and substitute into Eqt. 31 to obtain

$$\begin{aligned} f_{S,m}(s;m_0) = \sum_{n=m_0}^{m-1} & u_{m_0,n}[v_{n,m}(n+1,s) - v_{n,m}(n,s)] \\ & + u_{m_0,m}[v_{m,m}(s,s) - v_{m,m}(m,s)] \end{aligned} \tag{33}$$

where the constants of integration have cancelled.

Consider the indefinite integral from Eqt. 32

$$I = \int e^{-\lambda_n[t-n]} z_{n,m}(t,s)^{a-1} e^{-z_{n,m}(t,s)}\, dt \tag{34}$$

Applying a change of integration variable $\frac{dz}{dt} = -b_n$, using the fact from Eqt. 30 that $t - n = -\frac{z}{b_n} + \frac{B_{n,m}+b_m[s-m]}{b_n}$ and moving constant factors out of the integral, we obtain

$$I = -\frac{1}{b_n} e^{-\frac{\lambda_n}{b_n}[B_{n,m}+b_m[s-m]]} \int e^{\frac{\lambda_n}{b_n} z} z^{a-1} e^{-z}\, dz \tag{35}$$

$$= \frac{1}{b_n} e^{-\frac{\lambda_n}{b_n}[B_{n,m}+b_m[s-m]]} \left[\frac{b_n}{b_n - \lambda_n}\right]^a \Gamma\left(a, \frac{b_n - \lambda_n}{b_n} z_{n,m}(t,s)\right) + \text{constant} \tag{36}$$

Note that $\Gamma(a,z) = \Gamma(a)[1 - z^a \gamma^\star(a,z)]$ where $\gamma^\star(a,z)$ is the lower incomplete gamma star function. By using this form of the incomplete gamma function we remove the denominator $b_n - \lambda_n$ from Eqt. 36 which will cause numerical instability when $b_n \approx \lambda_n$. Substituting into Eqt. 36 gives

$$I = -\frac{\Gamma(a)}{b_n} e^{-\frac{\lambda_n}{b_n}[B_{n,m}+b_m[s-m]]} z_{n,m}(t,s)^a \gamma^\star\left(a, \frac{b_n - \lambda_n}{b_n} z_{n,m}(t,s)\right) + \text{constant} \tag{37}$$

Substituting this into Eqt. 32 gives

$$v_{n,m}(t,s) = -\frac{b_m \lambda_n}{b_n} e^{-\frac{\lambda_n}{b_n}[B_{n,m}+b_m[s-m]]} z_{n,m}(t,s)^a \gamma^\star\left(a, \frac{b_n - \lambda_n}{b_n} z_{n,m}(t,s)\right) \tag{38}$$

where the integration constant has been removed as it cancels in Eqt. 33. Also note that $v_{m,m}(s,s) = 0$ in Eqt. 33 because $z_{m,m}(s,s) = 0$.

Let $P_{m_0,m} = \int_m^{m+1} f_{S,m}(s;m_0)\, ds$ be the probability that a sheep introduced in month m_0 seroconverts in month m. Substituting in Eqt. 33 gives

$$\begin{aligned} P_{m_0,m} = \int_m^{m+1} \sum_{n=m_0}^{m-1} & u_{m_0,n}[v_{n,m}(n+1,s) - v_{n,m}(n,s)]\, ds \\ & - \int_m^{m+1} u_{m_0,m} v_{m,m}(m,s)\, ds \end{aligned} \tag{39}$$

Moving the integrals into the summation and moving constant factors out of the integrals gives

$$\begin{aligned} P_{m_0,m} = \sum_{n=m_0}^{m-1} u_{m_0,n} & \left[\int_m^{m+1} v_{n,m}(n+1,s)\, ds - \int_m^{m+1} v_{n,m}(n,s)\, ds\right] \\ & - u_{m_0,m} \int_m^{m+1} v_{m,m}(m,s)\, ds \end{aligned} \tag{40}$$

We define the indefinite integral

$$w_{n,m}(t,s) = \int v_{n,m}(t,s)\, ds \tag{41}$$

and substitute into Eqt. 40 to obtain

$$
\begin{aligned}
P_{m_0,m} = &\sum_{n=m_0}^{m-1} u_{m_0,n}\left[w_{n,m}(n+1,m+1) - w_{n,m}(n+1,m) - w_{n,m}(n,m+1) + w_{n,m}(n,m)\right] \\
&- u_{m_0,m}[w_{m,m}(m,m+1) - w_{m,m}(m,m)]
\end{aligned}
\tag{42}
$$

where the constants of integration have cancelled each other. Note that $w_{m,m}(m,m) = 0$. Substituting Eqt. 38 into Eqt. 41 we obtain

$$
w_{n,m}(t,s) = -\frac{b_m\lambda_n}{b_n} e^{-\frac{\lambda_n}{b_n}B_{n,m}} \int e^{-\frac{\lambda_n}{b_n}b_m[s-m]} z_{n,m}(t,s)^a \gamma^\star\left(a, \frac{b_n-\lambda_n}{b_n} z_{n,m}(t,s)\right) ds \tag{43}
$$

Applying a change of integration variable $\frac{dz}{ds} = b_m$, using the fact that $b_m[s-m] = z - B_{n,m} - b_n[n-t]$ and moving constant factors out of the integral, we obtain

$$
w_{n,m}(t,s) = -\frac{\lambda_n}{b_n} e^{-\lambda_n[t-n]} \int e^{-\frac{\lambda_n}{b_n}z} z^a \gamma^\star\left(a, \frac{b_n-\lambda_n}{b_n} z\right) dz \tag{44}
$$

The solution of which is

$$
w_{n,m}(t,s) = e^{-\lambda_n[t-n]} z_{n,m}(t,s)^a \left[e^{-\frac{\lambda_n}{b_n}z_{n,m}(t,s)} \gamma^\star\left(a, \frac{b_n-\lambda_n}{b_n} z_{n,m}(t,s)\right) - \gamma^\star\left(a, z_{n,m}(t,s)\right)\right] \tag{45}
$$

If a susceptible ewe, introduced in month m_0, tested negative in month m_- and then positive in month m_+ the probability of it doing so is

$$
p_+ = \sum_{i=m_-}^{m_+-1} P_{m_0,i} \tag{46}
$$

If a susceptible ewe, introduced in month m_0, never seroconverted until its last test in month m_r, the probability of it doing so is

$$
p_- = 1 - \sum_{i=m_0}^{m_r-1} P_{m_0,i} \tag{47}
$$

The equations for p_+ and p_- are found by applying Eqts. 30, 45 and 42. These equations are functions of the force of infection λ_i for each month i which, in turn, depend on the number of infectious ewes I_i in each month i. In the next section we describe how we find I_i.

Amparo et al. (2016) provides an algorithm to numerically solve the lower incomplete gamma star function.

1.3 Calculating the expected number of infectious ewes

In Peterson the number of infectious ewes equals the number of donor ewes which are assumed to be infectious from the start of the experiment until they are removed from their flocks in weeks 50 or 51. There are 10 donors in the Texels and 9 in the Blessumers (see Supplementary spreadsheet Peterson_data).

In Houwers the situation is more complicated because infected recipient ewes become infectious after a latent period. We use a discrete-time, infectious disease model to calculate the expected number of infectious ewes I_i, and the force of infection λ_i, in all months $i = 0, \ldots, M$ where M is the last test month.

We do not know the latent period L of an infected ewe. We therefore treat L as a Gamma distributed random variable with mean l and standard deviation σ. Let $F_L(y) = \Pr(L \le y) = P(y; {}^{l^2}/_{\sigma^2}, {}^{\sigma^2}/_{l})$ be the cumulative distribution function of latent period L, where $P(y; a, k)$ is the lower incomplete gamma function with shape a and scale k.

The contribution $I_{i,n+j}$, a susceptible ewe i, introduced into the flock in month $m_{0,i}$, infected in month n ($n = m_{0,i}, \ldots, m_{r,i} - 2$) and removed in month $m_{r,i}$, makes to the expected number of infectious ewes in

Parameter	Prior
β_{field}	Exp(0.02)
β_{housed}	Gamma(2, 0.1)
$\beta_{\text{unhealthy}}$	Gamma(2, 1)
τ	Uniform(1, 4)
l	Uniform(0, 60)
σ	Gamma(2, 1.5)
α	Uniform(0.001, 100)
μ_{healthy}	Exponential(24)
$\mu_{\text{unhealthy}}$	Exponential(24)

Table 1: Parameter prior distributions of full model.

month $n+j$ ($j = 1, \dots, m_{r,i} - 1 - n$) is the product of the probability the ewe is infected at time T in month n and the probability the ewe's latent period is less than or equal to $j - 1$ months, i.e.,

$$I_{i,n+j} = \Pr(n < T \leq n+1) \Pr(L \leq j-1) \tag{48}$$

$$= (\Pr(T > n) - \Pr(T > n+1)) \Pr(L \leq j-1) \tag{49}$$

Substituting in Eqt. 20 gives

$$= [u_{m_{0,i},n} - u_{m_{0,i},n+1}] F_L(j-1) \tag{50}$$

$$= u_{m_{0,i},n}[1 - e^{-\lambda_n}] F_L(j-1) \tag{51}$$

where $\lambda_n = \beta_n I_n$ is the force of infection in month n and $I_n = \sum_{i \in \text{ewes}} I_{i,n}$ is the expected number of infectious ewes in month n. Note that infectious ewes are assumed to have been removed in the last month they are tested and so do not contribute to the force of infection in that month.

We assume that donor ewe with ID 72 is infectious from the start of the experiment and so contributes 1 to the expected number of infectious ewes for each month until it is removed in January 1981. We test whether the other donor (ID 22) is infectious or latent at the start of the experiment.

1.4 Parameter prior distributions

When fitting to Houwers' data, we assign parameter prior distributions that are weakly informative reflecting the lack of information about the epidemiological parameters of SRLV transmission (Table 1). We use the mean parameter estimates from the fit of Houwers' data to assign the priors for fitting Peterson's data.

There are no published estimates of field transmission rates β_{field}. A weakly informative prior on field transmission rate can be found by assuming that all of the 23 of the 42 ewes that were infected over the 60 months of the experiment were infected by a single ewe whilst grazing. This is an overestimate because it neglects housed transmission and assumes a single infectious ewe was responsible for all infections. Assuming this gives a rate of $-1/60 \ln(1 - 23/42) \approx 0.01$. We take a slightly larger average rate of 0.02 to make the prior less informative. We assign an exponentially distributed prior—which has its mode at zero—as it is possible that field transmission might be close to, or even, zero.

There are numerous studies that suggest that housing is an important factor in SRLV transmission, but there are no published estimates of its value. We therefore assume that housing transmission rate is about 10 times the field transmission rate of 0.01. We therefore assign a weakly informative, Gamma distributed prior with shape 2 and scale 0.1 to β_{housed}, giving a mean transmission rate of 0.2 and zero probability of a zero transmission rate.

There are no published estimates of transmission rate in poor condition ewes, other than clinical observation that poor husbandry conditions and the presence of secondary infections are conducive to more

rapid spread of MV (Prichard and McConnell, 2007). We therefore assign a weakly informative, Gamma distributed prior with shape 2 and scale 1 to $\beta_{\text{unhealthy}}$, giving a mean transmission rate of 2.

Ewes were housed for at least one month during lambing in March and had access to housing for another three months from December to February. We therefore assign a flat uniform prior distribution from 1 to 4 to the duration of housing τ.

There are no published estimates of mean latent period l, or its variability σ. We therefore assign its mean l, a flat uniform prior between 1 and 60 months and its standard deviation σ, a weakly informative, Gamma prior with shape 2 (non-zero mode) and scale 15 months.

There is ample evidence that ewes seroconvert from a few weeks to several months after infection (e.g., Rimstad et al. (1993)). As discussed above, we have assumed that seroconversion period is Gamma distributed with shape parameter α and mean μ_{healthy} for healthy ewes and mean $\mu_{\text{unhealthy}}$ for poor condition ewes. As there is no prior information on the shape of this distribution we assign a weakly informative prior of Uniform(0.001, 100) to α. We assign exponentially distributed priors to μ_{healthy} and $\mu_{\text{unhealthy}}$ with a scale of 24 months.

The estimates from Houwers are used to assign the scale of the priors for Peterson (Table 2). In particular, the mean seroconversion period μ, is assigned a Gamma prior distribution with a mean of 8 months to reflect our inference about it from Houwers' experiment.

Parameter	Prior
β_{field}	Exp(8×10^{-4})
β_{housed}	Gamma(2, 0.1)
α	Exp(3.2)
μ	Gamma(2, 4)

Table 2: Parameter prior distributions for Peterson.

2 Calculating R_0 and the prevalence doubling time

Let $\mathbf{A}$ be a next-generation matrix whose elements $a_{i,j}$, are the expected number of ewes of age class j that are infected over the infectious lifespan of a ewe infected at age i Diekmann et al. (1990); van den Driessche and Watmough (2002). Let $\mathbf{N}_g$ be the vector of the number of infected ewes in each age class on generation g. Then, when MV prevalence is low, the number of newly infected ewes in the next generation is

$$\mathbf{N}_{g+1} = \mathbf{A}\mathbf{N}_g \tag{52}$$

The solution of this equation is $\mathbf{N}_g = R_0^g \mathbf{v}$ where R_0 is the dominant eigenvalue of $\mathbf{A}$ and $\mathbf{v}$ is its corresponding eigenvector whose elements are the stable proportions of infected ewes in each age class. The time between generations G, is the weighted mean ewe lifespan post infection. The weights are the stable proportions of ewes (infected plus susceptible) in each age class, and the average is taken over all age classes.

When MV prevalence is low, the growth equation of the total number of infected individuals is $|\mathbf{N}_g| = R_0^g|\mathbf{v}|$. The time since initial infection is $t = gG$. Substituting $g = t/G$ into this equation, taking logs and rearranging gives

$$t = \ln\left(\frac{|\mathbf{N}_g|}{|\mathbf{v}|}\right)\frac{G}{\ln R_0} \tag{53}$$

By setting $|\mathbf{N}_g| = 2|\mathbf{v}|$ and substituting into Eqt. 53 we obtain the prevalence doubling time $G\frac{\ln 2}{\ln R_0}$.

3 Markov chain convergence and mixing

In all models, three Markov chains were run for a total of 5×10^5 samples thinned to 5,000 samples with a burn-in of 5×10^5 samples. Markov chain convergence and mixing was assessed by examining (i) the marginal

distributions of each parameter of three parallel Markov chains (ii) the autocorrelation of each chain for each parameter and (iii) the Gelman-Rubin statistics of each parameter Gelman et al. (2003). Assessments are shown for the optimal model including field transmission for Houwers (Fig. 1), Peterson's Texels (Fig. 2), Peterson's Blessumers with (Fig. 3) and without (Fig. 4) the three outlying ewes.

References

Amparo, G., Ruiz-Antolin, D., Segura, J., and Temme, N. M. (2016). Algorithm 969 : Computation of the Incomplete Gamma Function. *ACM Transactions on Mathematical Software*, 43(3):26:1–26:9.

Cox, D. and Miller, H. (1965). *The theory of stochastic processes.* Methuen, London.

Diekmann, O., Heesterbeek, J. A. P., and Metz, J. A. J. (1990). On the definition and the computation of the basic reproduction ratio R0 in models for infectious diseases in heterogeneous populations. *Journal of Mathematical Biology*, 28(4):365–382.

Gelman, A., Carlin, J. B., Stern, H. S., and Rubin, D. B. (2003). *Bayesian Data Analysis.* Chapman and Hall/CRC, London, 2nd edition.

Gelman, A. and Rubin, D. B. (1992). Inference from iterative simulation using multiple sequences. *Statistical Science*, 7(4):457–511.

Prichard, G. and McConnell, I. (2007). Maedi-visna. In Aitken, I., editor, *Diseases of Sheep*, pages 217–223. Blackwells, 4th edition.

Rimstad, E., East, N. E., Torten, M., Higgins, J., DeRock, E., and Pedersen, N. C. (1993). Delayed seroconversion following naturally acquired caprine arthritis-encephalitis virus infection in goats. *American Journal of Veterinary Research*, 54:1858–1862.

van den Driessche, P. and Watmough, J. (2002). Reproduction numbers and sub-threshold endemic equilibria for compartmental models of disease transmission. *Mathematical Biosciences*, 180(1-2):29–48.

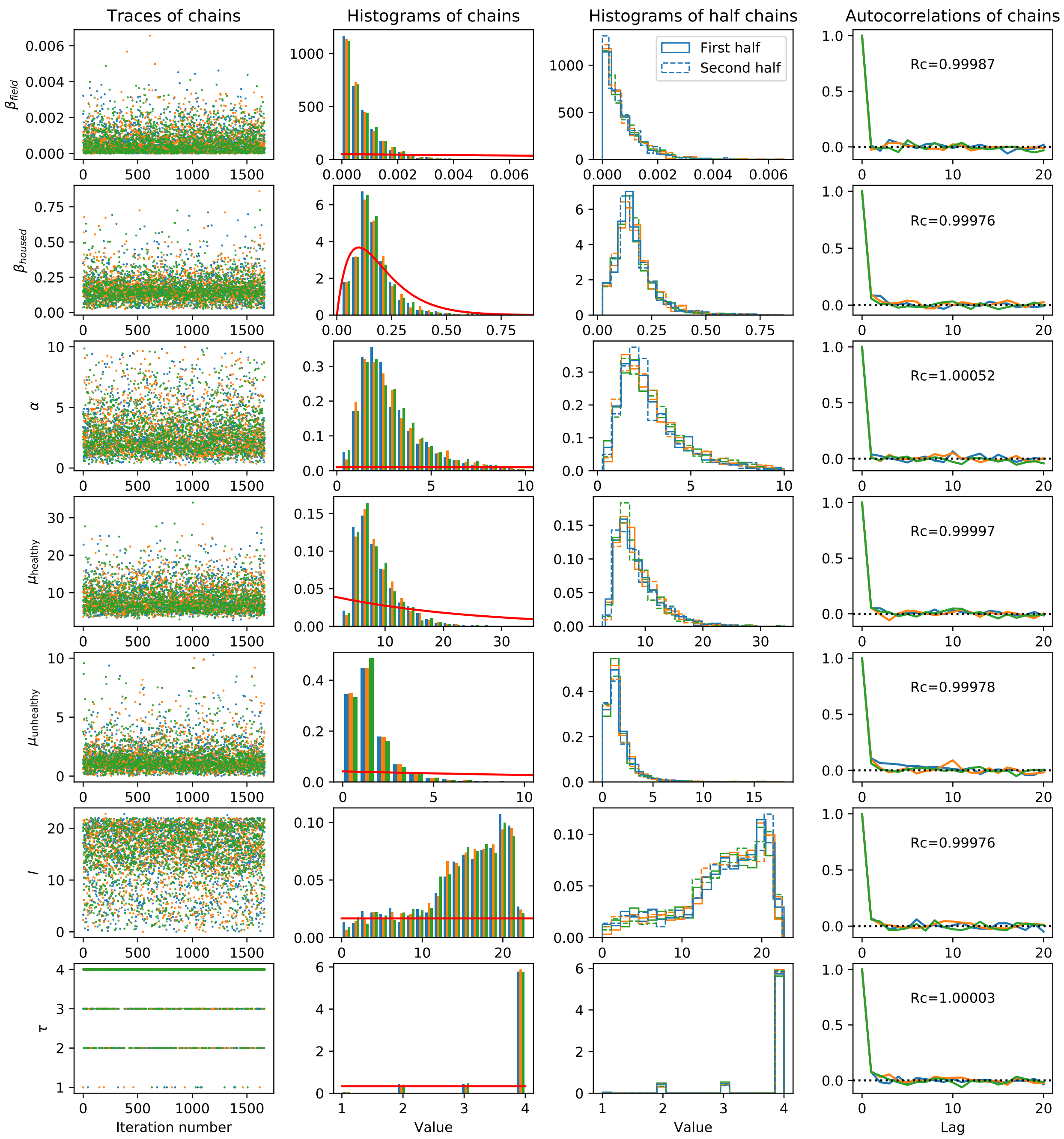

Figure 1: **Houwers.** Assessment of Markov chain convergence, mixing and autocorrelation for the optimal model including β_{field} for Houwers. The lefthand panels show the trace plots of three chains as green, blue and orange dots for each parameter. Their overlap demonstrates the chains' excellent convergence, stationarity and mixing. The similar chain histograms shown as green, blue and orange bars in the second column of panels demonstrate excellent convergence between the three chains. The overlapping histograms of the first (solid lines) and second (dashed lines) halves of each chain in the third column of panels demonstrates the chains' stationarity.The autocorrelation plots in the righthand panels demonstrate the lack of autocorrelation in the three chains. Gelman Rubin statistics (Rc, Gelman and Rubin (1992)) for each parameter is given in the righthand panels and demonstrate excellent convergence. Prior distributions are represented as red lines in the second column of panels.

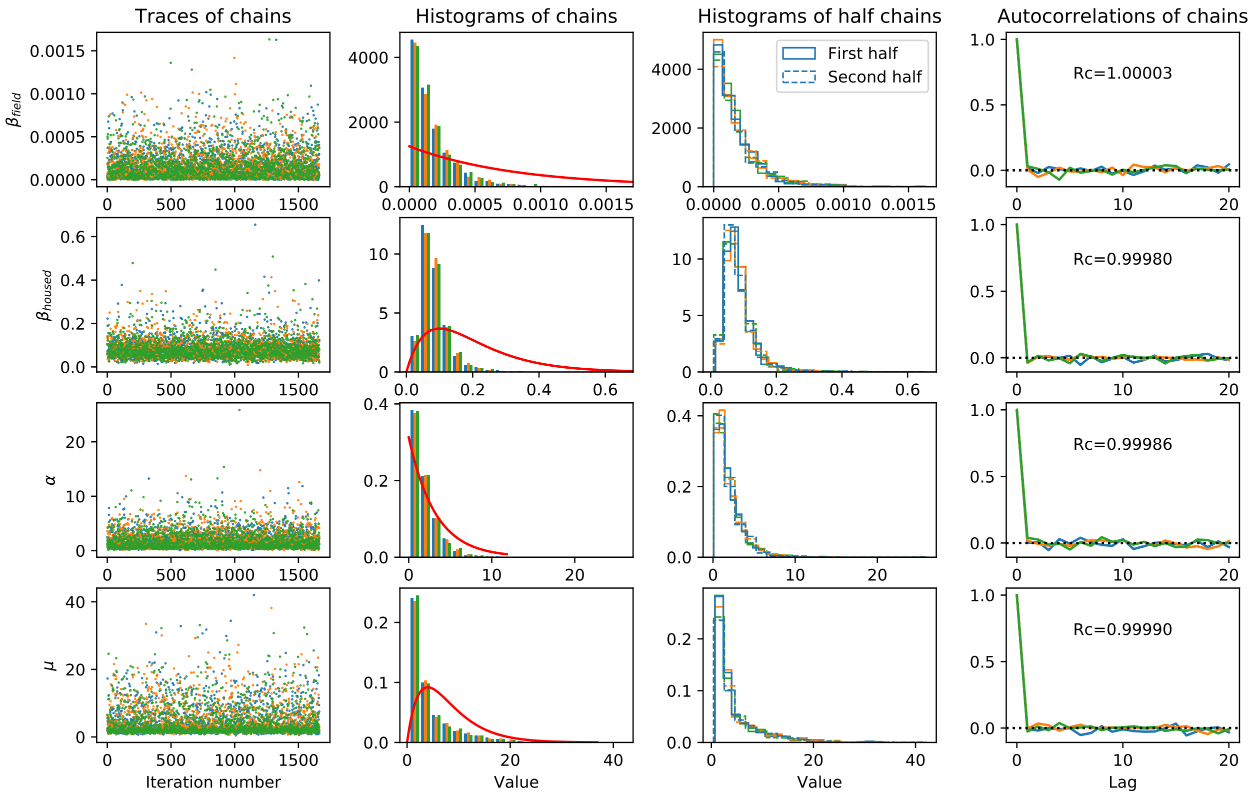

Figure 2: **Peterson's Texels.** See legend of Fig. 1.

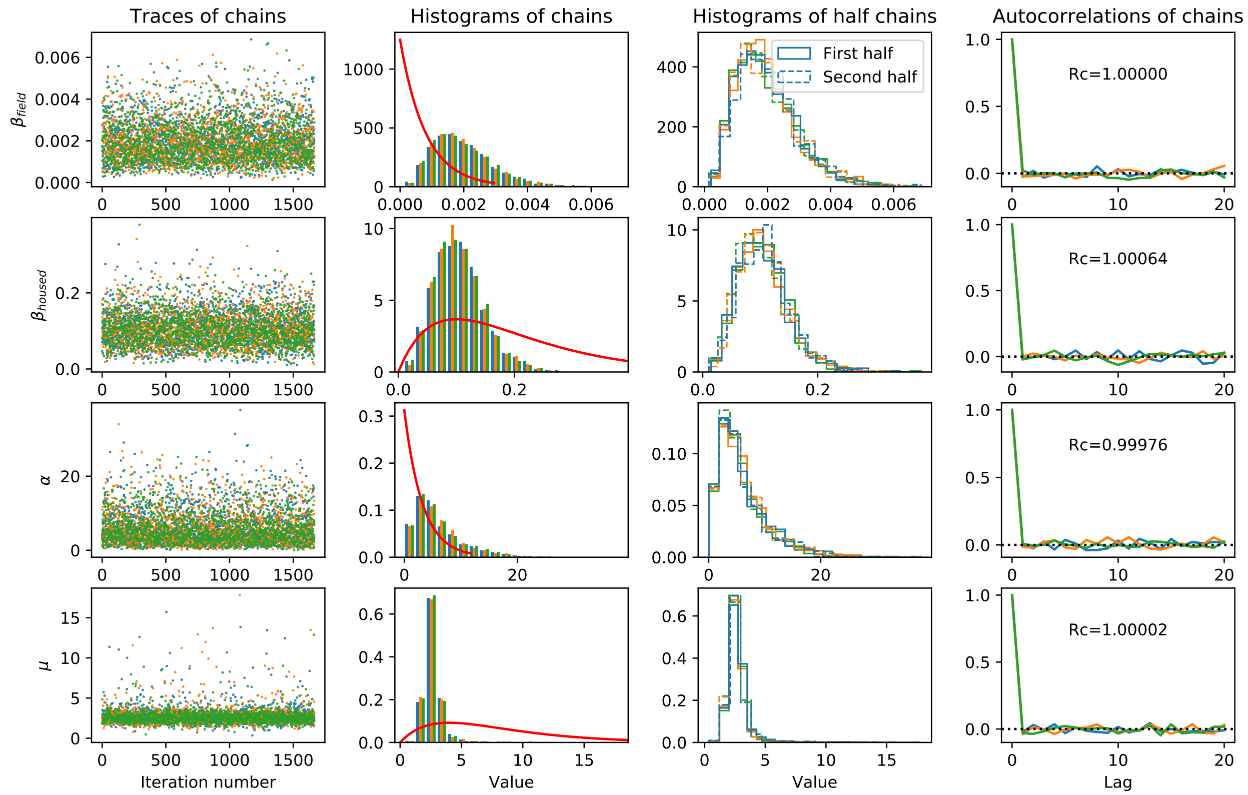

Figure 3: **Peterson's Blessumers with the three anomalous ewes.** See legend of Fig. 1.

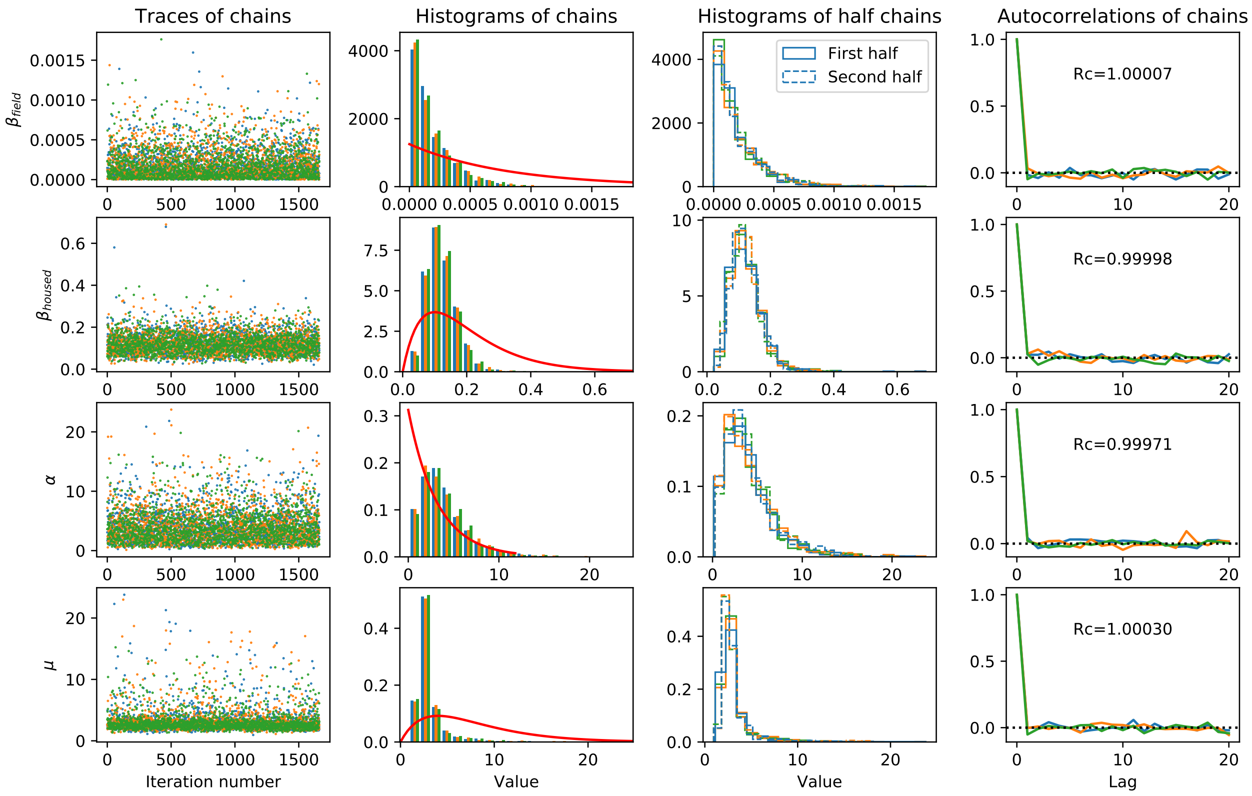

Figure 4: **Peterson's Blessumers without the three anomalous ewes.** See legend of Fig. 1.
