# Supplementary material for: Epidemiology and control of maedi-visna virus: Curing the flock
Source: PLoS One. 2020 Sep 10;15(9):e0238781. doi: 10.1371/journal.pone.0238781 (PMC7482963; doi:10.1371/journal.pone.0238781)
Supplement: Attachment — Submitted filename: Response_to_Reviewers.docx [file pone.0238781.s004.docx]

# Response to reviewers

## Reviewer #1:

**I found this to be an interesting and informative manuscript. However, I do have some concerns about the clarity of the model comparison done that I think should be addressed prior to publication. In addition, there are a number of typos throughout that could have been avoided by the use of a simple spell-check.**

We thank the reviewer for their careful reading of our ms and their comments which has greatly improved its clarity. Several typos have been corrected.

**By my count, the authors propose 720 potential models that reflect all possible combinations of 5 values of the number of resistant ewes, 2 levels of donor infectiousness, 4 effects of the winter of 1982, 3 levels of latency, 3 forms of transmission, and 2 shapes of the distribution of seroconversion periods (from Table 2). However, they only present results for 20 models. This implies that the authors had some a priori method of selecting from all possible model combinations. This should be described in the text. For example, did the authors select from all parameters using AIC to arrive at a model structure before selecting the number of resistant ewes? If so, what was the number of resistant ewes used to arrive at that initial model? If, as is possible, the values presented in table 2 are marginal AICs over all possible alternative models combining other parameters, then this needs to be described.**

AND

**L229 The authors should state what the preferred model is at this point. Simply stating the AIC makes the remaining comparisons confusing. Per the comment above about the full set of possible models, it is difficult to assess whether differences in individual parameters are the cause of better fit in the model comparisons below or if the bulk of the improved fit is driven by, for example, the change in the number of resistant ewes in the best fit model.**

In these comments the reviewer has pinpointed the confusion we have caused by not clearly separating out (a) the process of deriving and selecting a preferred, or optimal, model from (b) the testing of the model components to present evidence for its ability to explain the data, provide accurate parameter estimates and to be useful as a predictive tool. We have added a paragraph on model derivation and selection to the renamed subsection of the Methods "Model fitting, model selection and hypothesis testing", lines 201-207:

"Our model selection strategy was to derive a single optimal model based on infectious disease epidemiology and SRLV biology and judged by AIC~\cite{Akaike1974} and sum of squared residuals. Having derived the optimal model, we then tested each of its components by removing or adding the relevant parameters, refitting to data and examining the change in SSR or AIC and maximum likelihood. The optimal model thus provides the reference against which biological hypotheses are tested using model selection criteria."

We have also moved the description of the optimal model to the beginning of the Results and explained what it is we are presenting in the following analysis. Lines 246-255.

Fig. 4 has also been changed to show the fit of just the optimal model. The fits with 0 and 12 resistant ewes have been removed.

**L184-5 Authors state that convergence and mixing were assessed. These measures should be presented in the supplement so that readers can assess these on their own.**

The assessment of convergence and mixing of Markov chains with Gelman-Rubin statistics has been added Supplementary File S3 in Figs. 1-4.

**L188 The authors refer to “hypothesis testing” though really what is being done here is model selection. There are no formal null hypotheses or distributions of test statistics employed. This is a fairly trivial semantic point, but one that I would recommend should be changed. Throughout, the authors are simply choosing the best-fit model according to an information criterion and language used should reflect that.**

Is SRLV transmission more probable in housed conditions? This is a scientific hypothesis: an assertion to explain an observation that can be tested. We can test it by comparing the fits of two models - one with frequency dependent transmission and another with density dependent transmission (Cases 16 and 17 in Table 2) - to the data. Although we test the hypothesis using AIC rather than conventional null hypothesis significance testing (which is impossible with this dataset) it is, nonetheless, still a test of a scientific hypothesis. We understand that not everyone will agree with our use of the term "hypothesis testing", but we feel that this is what we have done and extend the new description of our procedure above to make this clear (lines 201-207).

**L324-327 This approach to removing 3 ewes to improve fit seems to come out of the blue and appears ad hoc. The authors should provide more justification for this choice. This seems analogous to the treatment of the resistant ewes for which the authors took a more formal approach. Is there not a parallel approach that could be applied here to address the influence of these 3 ewes? (Of course likelihoods are not comparable if you remove these 3 ewes, so one would need some change in parameterization to account for why these ewes are “ignored”).**

We agree that the removal of the three ewes is out of the blue. It is clear (given the data, what is known about SRLV epidemiology and the resulting parameter estimates) that these 3 ewes are outliers causing a poor fit and biasing the estimate of field transmission rate. It is therefore reasonable to remove them from the analysis and re-estimate the parameters. We could have estimated SSR as for the resistant ewes, but it is not necessary given the obvious better fit with them removed. We have re-written lines 352-361 to better justify the analysis with these 3 ewes removed:

"The data show that seroconversions were almost exclusively associated with the period after housing (Fig 6). The model fit to the cumulative seroconversions in the Texel experiment shows good agreement with the data (Fig 6A). The model fit to the Blessumer experiment is poor (Fig 6B) due to the three ewes that simultaneously seroconverted in the first week of housing. Their infection and seroconversion seem anomalous, because no other infections occurred during grazing. We therefore re-estimated model parameters after removing these three outlying ewes from the data. The model fit is substantially improved (Fig 6C). In the following comparison of parameter estimates we use the model without the three anomalous ewes."

We have also added the following on lines 371-374 to flag to the reader that a more in-depth discussion on the anomalous ewes is included in the Discussion section:

"In the model fit with the three anomalous Blessumer ewes, field transmission rate is biased upwards in order to account for their immediate seroconversion when moving from field to housed conditions (see Discussion)."

**L509 R0 should be defined earlier in the manuscript, when it is introduced.**

R0 was first mentioned in the Results. Instead we now introduce and define R0 in the Introduction as a key epidemiological parameter to estimate on lines 55-59.

**L547 “avoid” seems a strong statement here. I would soften this language. Perhaps this research suggests that MV may be a lesser problem, but I don’t think this goes far enough to justify that the problem would be “avoided”.**

We agree the statement should be softened to accommodate caveats about translation of our results to the real world, as follows on line 584-586:

"Our research suggests that extensive all-grass systems, which are increasingly being adopted in the UK for economic reasons, could avoid MV being a problem."

Our analysis of these experiments does clearly show that R0 would be less than 1 in non-housed groups of 100 ewes for our average estimate of field transmission rate (Fig 7B, and see related Discussion lines 557-567). *Under these conditions*, and if the experiments properly represented what occurs in wider practice, then an introduced infection would not spread, a pre-existing infection would die out, and the problems of MV would indeed be avoided.

**Table 3 — I would recommend including an comparison of the prior and posterior distributions of the parameters in the best fit model so that readers can assess the information on each of these parameters. It isn’t clear how many of these have moves substantially away from the prior.**

Parameter prior and posterior distributions of the optimal model have been added to Figs 1-4 in Supplementary File S3.

## Reviewer #2:

**Interesting piece of work modeling intra flock transmission of SRLV in sheep.**

We thank the reviewer for their kind comment.

**The viruses studied should be referred to as small ruminant lentiviruses and not MVV nor OPPV; it has been more than 20 years that those SRLV were demonstrated as heterogeneous and common to both sheep and goats (ann virol 1997 142: 1125 should be quoted); the interspecies transmission as quoted in ref 1 is misleading.**

We acknowledge the change in the nomenclature of SRLV and have made it throughout. In particular the first half of the first paragraph of the Introduction (lines 2-12) has been re-written:

"Maedi-Visna (MV) is an insidious, lifelong and eventually fatal disease syndromes in sheep and occurs in most sheep-keeping areas worldwide. The most common symptoms, chronic respiratory disease and indurative mastitis, only become evident some years after infection, and cause lost production and excess resource use. MV, called ovine progressive pneumonia (OPP) in North America, and its equivalent in goats (caprine arthritis encephalitis, CAE) are caused by the small ruminant lentiviruses (SRLV, family retroviridae), recognised as a heterogeneous group of viruses that infect sheep, goats and wild ruminants with evidence of cross-species infection (Leroux et al. 1997, 2010)."

**There is a major discrepancy between the potent mathematical tool and the small size of the 3 experiments of 20 -30 animals each.**

The epidemiological model is, in fact, quite simple: we use a standard SEI model in discrete time coupled with some basic probability theory to obtain the distribution of seroconversion times and hence a likelihood from which we infer parameter estimates using standard MCMC Bayesian methodology. The resulting optimal model has just 6 parameters (ignoring beta_field) for Houwers’ dataset and 3 for Peterson’s datasets. This number of parameters is reasonable given the number of data points (43 for Houwers and 20 for Peterson). We do not share the reviewers unsubstantiated opinion of a major discrepancy between model and data.

**The economic consequences of SRL infection quoted as a motto with references to reviews should be backed by research papers that are not existing to my knowledge.**

It is beyond dispute that the reduced welfare, longevity and productivity of animals infected by SRLV is economically costly. We could easily have expended a couple of paragraphs to evaluate it, based on published research, had it seemed necessary.

**The horizontal transmission has been suggested as early as beginning of this century (see Vet Res 2004 35: 257; paper that review correctly SRLV infection.**

Actually, Icelandic evidence from the 1950’s suggested horizontal transmission. But the important point is not the date but that most reviews (including Peterhans et al Vet Res 2004 35: 257) emphasise the importance of vertical, and particularly lactogenic, transmission. We show this to be epidemiologically irrelevant, which rather updates the significance of horizontal transmission.

**There is a strong issue regarding the property of the original datas also the so called Peterson original set of data seems accessible this is not the case of the so called houwers dataset. The origin, accessibility and ownership of the original experimental datas should be stated.**

Contrary to the reviewer’s comment, the full Houwers dataset is published in Houwers and van der Molen (1987) J. Vet. Med. It is therefore accessible and in the public domain and there is no issue with ownership. The Peterson dataset is owned by Karianne Lievaart-Peterson who is an author of this study, so again, there is no issue with ownership. Contrary to the reviewers comments we have cited the relevant studies (Houwers et al. J. Vet. Med. (1987) and Peterson et al., Utrecht University (2007)), and provided the datasets as Supplementary Material.

**Reading through dr Peterson thesis it seems she intended to publish a similar analysis to the present manuscript based on what is her own results . What is the status of this manuscript (Peterson K., Stegeman J.A., Colenbrander B., Houwers D.J. Quantification of the horizontal transmission of maedi visna virus between sheep in two different breeds. Submitted for publication) see ref 12 p127 of the thesis.**

The purpose of the study in dr Peterson’s PhD thesis, cited as **"**Peterson K., Stegeman J.A., Colenbrander B., Houwers D.J. Quantification of the horizontal transmission of maedi visna virus between sheep in two different breeds. Submitted for publication", was to distinguish breed differences in susceptibility. As it failed to find any such differences publication was not pursued.

**Furthermore since all 3 experiments are from dr Houwers team how come he is not a coauthor. The reviewer has major concern regarding appropriation of datas and ideas.**

As Prof. Houwers data is in the public domain it is not obligatory for him to be a coauthor. However, out of courtesy, and to gain his insight into his experiment, we contacted him by phone and email. He was unable to recollect any information other than what was published, he did not contribute to the analysis and interpretation of our work. We did, however, neglect to acknowledge Prof. Houwers. We have now rectified this oversight on line 608.

**The conclusions are not backed by the observations and the analysis, the experimental conditions of the 3 flocks were outside and inside.**

Our analysis is able to distinguish SRLV transmission rate when sheep were inside from that when they were outside. It is legitimate to apply our estimate of field transmission rate to predict the epidemiological consequences of keeping sheep only at grass, and avoiding housing. The disease transmission that occurred during grazing in the three experiments we analysed was not sufficient to cause disease incidence to increase (R0>1). Houwers and van der Molen (1987) made the observations that “*The majority of seroconversions took place in the first halves of the years, which suggests that the horizontal transmissions mainly occurred in the winter seasons. This underlines the probable epidemiological importance of housing*”. The absence of transmission during grazing is quite obvious in the Peterson experiments (see Fig 6). Therefore, our conclusions are entirely consistent with the observed epidemiology and our analysis.

**The concept of non infected animals is naive when seronegative animals can be µPCR positive in the blood or in the semen (see Therionelogy 2008 69: 433. Thus the first assumption (namely that the rams were not infected) is not supported.**

We agree that ‘non-infected’ is a dubious status, but it is fair to assume that rams from uninfected flocks will themselves be uninfected. That is what makes accreditation schemes a success. Houwers and van der Molen (1987) states: "*Each year, a ram was added from a flock which had been free of seropositive sheep for at least the preceding three years*.". So the assumption that rams were not infected is well supported by Houwers’ own statement.
