# Supplementary material for: Epidemiology and control of maedi-visna virus: Curing the flock
Source: PLoS One. 2020 Sep 10;15(9):e0238781. doi: 10.1371/journal.pone.0238781 (PMC7482963; doi:10.1371/journal.pone.0238781)
Supplement: Attachment — Submitted filename: Response_to_Reviewers.docx [file pone.0238781.s005.docx]

# Response to reviewers

I agree with other Reviewers that clarity in the model comparison must be improved prior to publication and the comments on the use of the term hypothesis testing which has a specific meaning from statistics that I am not sure is intended here. I was expecting statistical hypothesis testing to appear.

- **The Results section has been extensively re-written to avoid the terminology of null hypothesis statistical tests and instead use the terminology of model selection using the information-theoretic approach of Burnham and Anderson (2002).**

If an economic analysis with cost benefit ratio or similar (or a least an estimate of annual losses at some appropriate geographic/geopolitical/global unit) is available it could easily be added in a phrase or sentence without expanding too much.

- **We have made reference (Gibson et al., in the Introduction) to an analysis of costs of controlling MV (line 15).**

**Abstract**

- Background, methods, results well included but could be modified to close with some conclusions and future directions. Many results are just listed and might be helpful to put in context. Ending could be made up of the last sentence (or two) of the Introduction, which sound more like they belong somewhere in the middle and end of the abstract.
  - **We have expanded the text to provide further context. We feel that the last two sentences at the end of the Introduction are best left there to round it off.**
- The latency sentence does not clearly describe your finding due to the punctuation. Instead of colon, consider putting the definition between commas or in parenthesis from “a time… …infectiousness”.
  - **We have changed this sentence to “We demonstrate the existence of epidemiological latency, which has not explicitly been recognised in the SRLV literature. This time delay between infection and infectiousness apparently exceeds the delay between infection and seroconversion.”**
- What do you think the mechanism behind poor body condition being associated with more rapid seroconversion is? Interesting finding. You explain this in the Discussion but you may be able to hone abstract enough to discuss a few of the results points briefly here.
  - **We feel it would be unwise to speculate on this point in the Abstract, and prefer to leave this to the Discussion.**
- Replacement lambs – what exactly does this term mean? From the abstract it’s not clear why there is a low probability of them being infected but from the paper this is a clearly stated result. Make this sentence sound more like a result here in the abstract. “Maternal transmission was found to have a small role in transmission, as replacement lambs from infected ewes had a low probability of being infected directly by their mothers.” The slight changes make this sound more like a finding from your study not general knowledge.
  - **We have changed “replacement lambs” to “lambs need be retained to maintain flock size”.**
  - **We have clarified the sentence about maternal transmission: “Maternal transmission has only a small role in transmission, because lambs from infected ewes have a low probability of being infected directly by them, and only a small proportion of lambs need be retained to maintain flock size.”**
  - **He have added the sentence “Our results show that MV is overwhelmingly a disease of housing, where sheep are kept in close proximity. ” to reinforce our main conclusion.**

**Introduction**

- Lines 3-5 – citation?
  - **We have inserted a reference to Blacklaws 2012 (line 5).**
- Line 9 – what other species are often infected with SRLVs?
  - **We feel it is sufficient to provide references for this (Leroux et al. 1997, 2010), rather than a list of affected wildlife species (line 9).**
- Line 13-14 – best split into two sentences
  - **Done (line 15).**
- Line 17 – do or do not? Unclear
  - **We have clarified this sentence by removing the word "also" from “… do not also target …” (line 19).**
- Line 21 –on average, how long is seroconversion delayed?
  - **We have specified a typical seroconversion delay (lines 26-27) and re-ordered the second paragraph to improve flow. Seroconversion delay is dealt with at greater length in the Discussion.**
- Suggestion: Hone and polish this section a little further by removing unnecessary words.
- Comment: Last two paragraphs are a little choppy due to shorter sentences and a lot of information.
  - **We believe we have the right balance between shortening and lengthening the text.**
- Last few sentences sound like they belong in the middle (copied ‘We applied our…) and end of the abstract (moved not copied ‘Our results provide…’) instead of here at the end of the Introduction.
  - **We feel these sentences are best left at the end of the Introduction to round it off, as is normal.**

**Methods**

- “and some other ewes were removed.” – unclear
  - **We have reworded to “Some other ewes were removed to maintain flock size in the range from 21-37 over the experiment” (lines 84-85).**
- Data set descriptions: Please include the manufacturer of the indirect ELISA kit, what is the sensitivity and specificity of the kit for MV virus in each of the species tested.
  - **These are now included on lines 80-81 for Houwers and lines 96-97 for Peterson.**
- What would be the proposed mechanism of resistance to MV infection? Resistance and what is known about the mechanisms should be described in the Introduction as this seems to be the first time it is considered in detail. The answer to this question appears to be done later in lines 206-210. Perhaps this should be earlier.
  - **To anticipate resistance to MV infection in the Methods, we now state in the Introduction “Breed differences in susceptibility and the genetics of resistance to SRLV are recognised” with two references (lines 9-10).**
- Assumptions section – first four bullets are clearly assumptions. Subsequent bullets mix assumptions and what was tested. Perhaps keep this section assumptions and then have what was tested in another section of bullets? This is a really long block of bullets and not all text is assumptions.
  - **For clarity, we now list just assumptions of the full model (which contains the full set of parameters and is the model derived in the Supp. Info.) rather than list a mixture of fixed and tested assumptions (lines 112-146).**
- Is this a reasonable assumption? Do you think the empirical data suggest variability in ewe infectiousness? Was sensitivity analysis conducted of a few example situations in which ewes are not equally infectious and in which infectivity does not remain constant (use various patterns over time) to see the impact on model dynamics? Anything from the literature on this?
  - **There is insufficient data to determine if infectiousness varies between ewes and varies over the duration of infection. There is no qualitative let alone quantitative evidence in the literature.**
- Did you test transmission scaling intermediate between the extremes of density dependence and frequency dependence? This has been done in other systems such as in Smith et al 2009 PNAS (Host-pathogen time series data in wildlife support a transmission function between density and frequency dependence) and Cross 2013 Ecology (‘Female Elk contacts are neither frequency nor density dependent’) If you did not and no plans to, maybe discuss.
  - **Again, there is insufficient data to examine transmission scaling intermediate between the extremes of density dependence and frequency dependence. The datasets from Smith et al 2009 and Cross 2013 are at least an order of magnitude larger than Houwers and Peterson.**
- What is the impact of your assumption of varying which months were on pasture in the face of the lack of data on duration of housing each month?
  - **There is no discernible impact on parameter estimates as the estimate of the duration of housing was quite broad and no covariance with other parameter estimates was found. In addition, we know precisely when ewes were housed in Peterson, and because of the similarity in parameter estimates between the two datasets we saw no need to discuss this further.**
- For latent and seroconversion period, why were those distributions (exponential and gamma) assumed and why were the specific distributions tested? This is mentioned a bit in 156-159 and might be nice to have these closer together.
  - **The justification for the Gamma distribution has been moved into the bullet point about this specific assumption (lines 140-142).**
- Bullet at bottom of page 4, lines 138 – 142. This is unclear and although it contains and assumption and a test starts to be part of something that perhaps should be prose?
  - **We have removed this bullet, rewritten it, and added it to the main text on lines 146-152: “In Houwers, twenty-four ewe lambs were retained within the flock. Eighteen of these lambs were born to seronegative dams. One was born to a donor ewe and was vertically infected by its dam. We do not model this infection event, but instead assume this lamb was infected at birth. The other five lambs were born to seroconverted dams and seroconverted at least 14 months after birth. Given this long interval between birth and seroconversion, we assume that these lambs were infected horizontally rather than vertically.”**
- Line 147: So there is little to no maternal immunity? What is known on maternal immunity should be briefly included in Introduction. So far I only see in the Abstract and here. Later on I find in the caption to Figure 7 with a Reference. And then a nice section in Discussion. Having a hint more mention in the beginning would be helpful.
  - **We believe when the referee talks about “maternal immunity” they mean maternal antibodies passed from dam to lamb which confer immunity to the lamb. This is a different issue to maternal transmission of the virus which, as the referee says, we talk about in the abstract, discussion and the caption in Fig. 7. The referee's comment though made us realise that we had not considered maternal antibodies and that our assumption on line 147 might not hold. We re-examined the data and came to the following conclusion. Six of the 24 lambs born were born to seroconverted dams. These lambs were tested 2 to 4 months after birth and none was seropositive. So, it is not possible to tell if they received maternal antibodies given that maternal antibodies wane in the first few months of life. In the model we assume that these lambs do not have maternal antibodies and so are fully susceptible from birth. To examine this assumption, we re-fitted the optimal model assuming that these lambs were protected for the first two months of life. This resulted in no change in model fit or parameter estimates. We have added the justification for ignoring maternally-derived immunity on lines 152-157.**
- Line 167 – should this be ‘up to three’?
  - **As we now detail the full model in the Methods, this sentence has been re-written to say “specified by four constant parameters” (line 167).**
- Line 194 – spell out AIC first time and give acronym for SSR here ahead of it’s usage a few lines later
  - **Done (lines 226-227).**
- Please explain how you will definite a significant enough difference in SSR or AIC to differentiate between models.
  - **As we do not do null hypothesis significance testing we cannot apply a significance threshold. Instead we use changes in likelihood and AICc and the evidence ratio (Burnham et al 2011) to quantify the evidence for the optimal model. It is important to note that we do not just rely on changes in AICc and SSR as evidence for the optimal model. We also rely on evidence from Houwers' 1987 paper and the wider literature to inform and justify it.**
- Table 1 – why is field transmission exponential but household transmission rate gamma distributed? Suggest explaining in text.
  - **This is explained in Supplementary Information Section 1.4. As there is prior evidence in the literature that transmission in the field is less likely than transmission in housing, it is possible that field transmission might be close to zero. The Exponential prior, which has a mode at zero, allows for this in the posterior distribution, which the Gamma prior does not. We have removed the priors from Table 1, and included a new Table (Table 1) in the Supplementary Information , as they detract from the narrative in the main text.**
- What language and/or programs was the model coded in and what version?
  - **We have added that the code is written in C on line 201.**
- Unclear how determining resistant ewes will be used later in this analysis or for providing inference for control.
  - **The resistant ewes are not used later in the analysis as (i) it is impossible to determine if there were resistant ewes in Peterson's experiment (line 176-177) and (ii) they do not contribute to R0. We have, however, added that the number of ewes in the R0 analysis only include susceptible ewes (lines 248, 253, 390, 392, legend of Fig. 7).**
- Line 220: How many age classes in model?
  - **The model has 66 age classes each 1 month long to accommodate a latent period of 15 months (line 249).**

- Lines 226-232: suggest adding some results (in form of figure, etc) to the supplementary information so reader can make comparison. It is a bit unclear what occurred in lines 226 to the end of the section so hard to assess appropriateness of methods.
  - **The sentences describing the corroboration of the results of next generation model with the results of the deterministic and individual-based models have been removed. The corroboration was for our own peace of mind and are not required for this paper.**
- Line 234: cite appropriate references for Next Generation Matrix (example van den Driessche etc)
  - **Diekmann et al 1990 and van den Driessche and Watmough 2002 have been added (line 247 and in the Supp. Info.). We apologise for this oversight.**
- Overall, methods could benefit from a little more information on the mathematical model that is instead located in the supplement.
  - **As the paper’s main intended audience is the SRLV community (who are, for the most part, not mathematically trained) we chose to move the mathematical details of the model to the Supplementary Information.**

**Results**

- This is the most challenging part of the paper for the reader, as it is hard to follow and to know what is important finding and interesting finding. Some of the text appears to be a Discussion of the results. Figures are fine (but wish they were located by their captions).
  - **The Results section has been extensively re-written and clarified.**
- Houwers 2. Donor infectiousness – need to explain what a difference of around 3 in AIC means for decision making. Is this strong evidence or not? While the smallest AIC was usually selected for several comparisons, the difference in AIC was not large and what each difference means for how different each model is should be better explained in Methods and Results.
  - **As suggested by Burnham et al. (2011), we now use ΔAICc and evidence ratio as the method to quantify the evidence for the components of the optimal model. Table 2 has been modified to include these new values. ΔAICc and evidence ratio are introduced and explained in the Methods (lines 238-241).**
- Also explain why you make the conclusion in line 281.
  - **We have clarified this conclusion on lines 310-315.**
- Overall, am not sure if the test in 2 and 3 are needed for this paper. It seems the main findings and take aways are about the distribution of seroconversion time, impact of housed vs pasture management, etc. Yet the Methods and Results keep being listed in order. Perhaps some restructuring of main findings vs additional findings would help flow for reader. Table 2 does help make it easier to follow results in the Houwers model section.
  - **We have re-ordered the evidence for each of the model's components in the main text (lines 262-267) and Table 2 to reflect their importance.**

- “density-dependent transmission, in which field and housed transmission rates are independent” – this was an unexpected definition of DD transmission (can’t densities also be high on the field?). Please clearly state your definitions of the transmission scaling types in the Methods. Some of the text of this Results section belongs in the Methods so just the Results are reported in Results.
  - **We use the definition of frequency and density dependent transmission as per Begon et al. 2002 (reference now included on line 120). Stocking density varies by several orders of magnitude between field and housed conditions. Although densities in the field can change they do not approach densities when ewes are housed.**
- Is the AIC difference between frequency and density dependence sufficiently large to say there is an important difference? Same question with section 6 Distribution shape. Are the differences large enough to conclude something?
  - **In Houwers the evidence is not large (1:4.2) because the peak in seroconversions in 1982, explained by an increase in seroconversion rate due to poor body condition, partly masks infections in housing that winter (now explained on lines 307-309). However, the evidence from Peterson is much stronger (1:190, Texels and 1:309 in Blessumers). These results are now included in a new Table 4 and on lines 374-377.**
- “Given the assumption that seroconversion period is Gamma distributed, this means that it may take roughly 1.5 years for 95% of healthy ewes to seroconvert and 2.5 months for 95% of ewes with poor body condition.” This calculation is unclear, please explain further. Lines 316-318.
  - **We use the gamma distribution's quantile function at 0.95 with mean parameter estimates of the optimal model to calculate these values (line 353).**
- Lines 364-365:” months for R0 values in typical commercial flocks that house tens of ewes together over winter and for lambing” – citation? What is the range of those R0s? Can they be highlighted on Figure 8? Also some of the paragraph seems like explanation that belongs in Discussion.
  - **An additional sentence has been added for commercial flocks: “For commercial flocks which house large groups of ewes together for several months over winter and for lambing, $R_0$ is predicted to exceed 10.” (Lines 393-394). This clarifies the sentence about doubling time which has also been changed: “For commercial flocks, with predicted $R_0$ greater than 10, doubling time is expected to be about 6 months.” (lines 403-404).**
- What are the main findings from the Results? At the end I am a bit confused, suggesting restructuring could help the reader follow the arguments and focus on important findings, their meanings, and implications for control. Takeaways become much clearer in Discussion.
  - **We feel the restructuring and re-writing of the Results has clarified this section, stating, in particular, the main results at the beginning of the results section.**

**Discussion**

- Latencies – some of this information sounds more like it belongs in Introduction.
  - **There is no useful introductory material on latency in the MV literature because its existence has not, apparently, been suspected.**
- Line 392: cv?
  - **“cv” means coefficient of variation. Added on line 431.**
- Line 472: based on the findings before it – is this further research needed (sounds mostly like things that were covered already by previous research)? If not, what else is missing that you would recommend?
  - **The literature is rather weak on what housing characteristics might affect transmission. We have re-written this sentence to make it clear that further research might turn up ways to mitigate the effects of housing on transmission: “There is rather limited information on precisely what aspect of housing---such as the proximity of sheep, the shed's ventilation characteristics, the sharing of drinkers and feeding areas---are most associated with SRLV transmission. Further research could indicate whether re-designing sheep housing would have merit in controlling transmission.” (lines 518-522)**
- Line 518: Perhaps including the R0 equation for this model system earlier in the paper might be helpful. (also see comment below on supplemental information).
  - **There is no equation. R0 is numerically determined from the next generation matrix using the GNU Scientific library. A new reference has been added at line 256.**

**Conclusions**

- Line 547 – synthesize?
  - **We use the British spelling of synthesise.**
- Line 556 – Suggest rewording last phrase
  - **The sentence reads well to us, and we have already toned down this sentence from previous versions.**
- Does second paragraph belong in Discussion and not in the Conclusions?
  - **We feel this paragraph concludes our findings well so we will leave it as is.**

**Supplemental**

- Pg 1 equation 2: what is the o in o(delta t)?
  - **o(delta t) is little-o notation. However, the derivation of the probability density functions of infection time and seroconversion period have been updated and do not now use o(delta t).**
- Where is the derivation of the R0 (or was it simply only obtained from next gen matrix)?
  - **R0 is numerically determined from the next generation matrix.**
- Add references for next gen matrix to section 2 or reference list.
  - **References Diekmann et al. 1990 and van den Driessche and Watmough 2002 have been added.**
